# Primary ovarian leiomyoma with calcification: A case report

**DOI:** 10.1097/MD.0000000000039122

**Published:** 2024-07-26

**Authors:** Dajiang Lu, Hua Chen, Chengfang Yang, Yanzhen Liu, Haishan Mo, Yongguo Zhang

**Affiliations:** a Department of Gynecology, the People’s Hospital of Qiannan, Duyun, Guizhou, P.R. China; b Department of Obstetrics, the People’s Hospital of Qiannan, Duyun, Guizhou, P.R. China; c Department of Plastic Surgery, the People’s Hospital of Qiannan, Duyun, Guizhou, P.R. China; d Department of Anesthesiology, the People’s Hospital of Qiannan, Duyun, Guizhou, P.R. China.

**Keywords:** calcification, case report, diagnosis, primary ovarian leiomyoma

## Abstract

**Rationale:**

Primary ovarian leiomyoma is a rare benign tumor. The exact histological origin and pathogenesis of primary ovarian leiomyoma are still unclear, while its preoperative imaging diagnosis is often challenging and prone to misdiagnosis. The study aims to elucidate the diagnosis of primary ovarian leiomyoma and to distinguish it from fibroma.

**Patient concerns:**

A 34-year-old female was admitted to the hospital with complaints of pelvic mass found for one year. One years ago, the patient went to a local hospital for examination due to irregular menstruation.

**Diagnoses:**

The ultrasound report of the patient showed ovarian teratoma. The postoperative pathological results showed ovarian leiomyoma and calcification.

**Interventions:**

The patient underwent laparoscopic right ovarian leiomyoma resection.

**Outcomes:**

The patient was discharged home three days after surgery. At the most recent follow-up (five months after operation) of the patients, ultrasound was performed and no abnormal echoes were suggested in the adnexal region.

**Lessons:**

In the diagnosis of primary ovarian leiomyoma, our case emphasizes the importance of microscopic features as an effective approach to distinguish it from ovarian fibroma, leiomyosarcoma, and stromal tumors. Additionally, personalized treatment should be considered based on the patient age and fertility needs.

## 1. Introduction

Primary ovarian leiomyoma is a rare benign tumor, accounting for 0.5% to 1% of all benign ovarian tumors.^[1]^ It most commonly occurs in women aged 20 to 65 years and is asymptomatic in most cases, usually discovered by chance during routine gynecological examinations and surgeries.^[2]^ Though it is generally believed that it may arise from smooth muscle cells in the ovarian hilar blood vessels, its clinical features and tissue origins are currently not well understood. Moreover, because of its similarity to fibromas, it can be easily misdiagnosed.^[1,2]^ Herein, we present a case of primary ovarian leiomyoma with calcification diagnosed in a 34-year-old female in our hospital to elucidate the diagnosis of primary ovarian leiomyoma and to distinguish it from fibroma. The article followed the guidelines of the Helsinki Declaration and was approved by the ethics committee of the hospital. The article also complies with the CARE reporting checklist.^[3]^

## 2. Case presentation

A 34-year-old female was admitted to the hospital with complaints of pelvic mass found for 1 year. One years ago, the patient went to a local hospital for examination due to irregular menstruation. The last menstrual period (LMP) of the patient was July 12, 2022, with regular menstruation. The ultrasound report showed an ovarian teratoma, and the doctor advised her to undergo surgery, but she did not follow the doctor advice. For further confirmation, she received examination our hospital on July 18, 2022, and the ultrasound report showed ovarian teratoma (a mixed mass in the right adnexa). The patient was hospitalized in our department and diagnosed with hypertension and type 2 diabetes. However, due to poor control of blood sugar and blood pressure, surgery could not be performed in time. When blood pressure and blood sugar control stabilized, the patient visited our hospital again.

**Figure 1.**
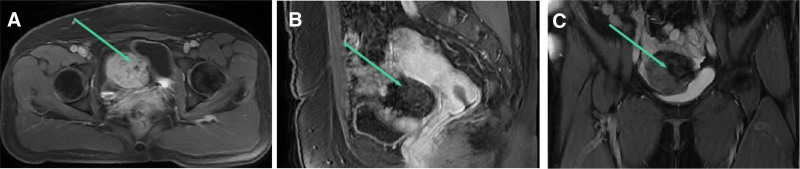
MRI findings. (A) The cross-sectional enhanced scan showed that most of the lesions showed significant enhancement, with no patchy or unenhanced areas visible, and the lesion boundaries were clear; (B) The sagittal position of T1 enhanced scan shows a patchy unenhanced area in the upper part of the lesion, while a clearly enhanced area can be seen in the lower part of the lesion, and the boundary between the posterior part of the lesion and the uterus is clear; (C) T1 enhanced coronal scan shows a patchy unenhanced area in the upper left part of the lesion, with obvious enhancement visible in the right part of the lesion. The boundary between the upper right and the right appendix is unclear.

Gynecological examination showed that the pubic hair was evenly distributed; the vagina was unobstructed, with a small amount of white discharge inside, and no odor; the cervix was enlarged, medium in quality, without contact bleeding, and without pain when lifting; anterior uterus, normal size, no tenderness; a 6 × 5 cm mass was palpable in the right adnexal area without tenderness, and no mass or tenderness was palpable in the left adnexal area. Gynecology ultrasound revealed a cystic solid mass with a diameter of about 6 cm in the right adnexal area, movable, no obvious tenderness, and no obvious abnormality in the left adnexal area. Laboratory examinations showed that the levels of carbohydrate antigen 125, carbohydrate antigen 199, carcinoembryonic antigen, and Alpha-fetoprotein were 28.01 U/mL, 10.54 U/mL, 1.31ng/mL, and 1.83 ng/mL, respectively. MRI demonstrated ovarian teratoma (Fig. 1).

**Figure 2.**
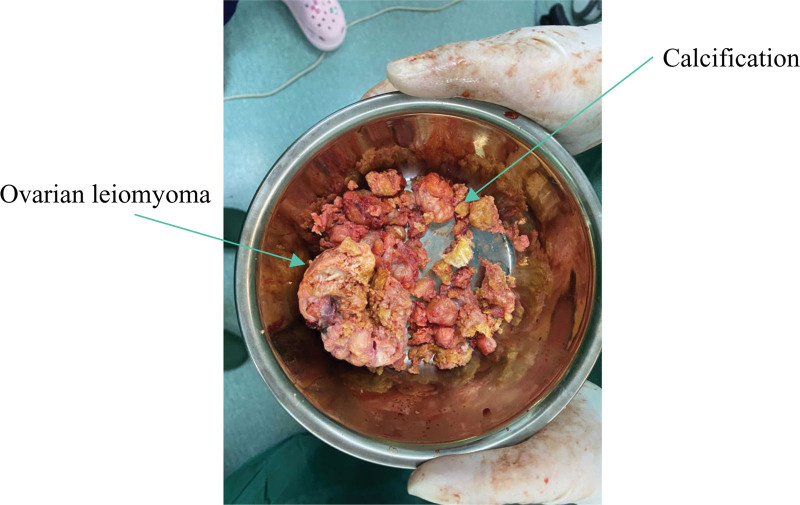
Gross pathology photo of surgical specimen.

The patient underwent laparoscopic right ovarian tumor exfoliation on July 22, 2022. The cortex of the right ovarian tumor was laparoscopically cut with scissors to make an incision about 0.3 cm in size, along which the right ovarian tumor was peeled off and completely removed, and then the tumor was put into a collection bag for extraction. Due to the hard texture of the tumor, the puncture hole in the left lower abdomen was enlarged, and the tumor was taken out and sent for pathological examination (Fig. 2).

**Figure 3.**
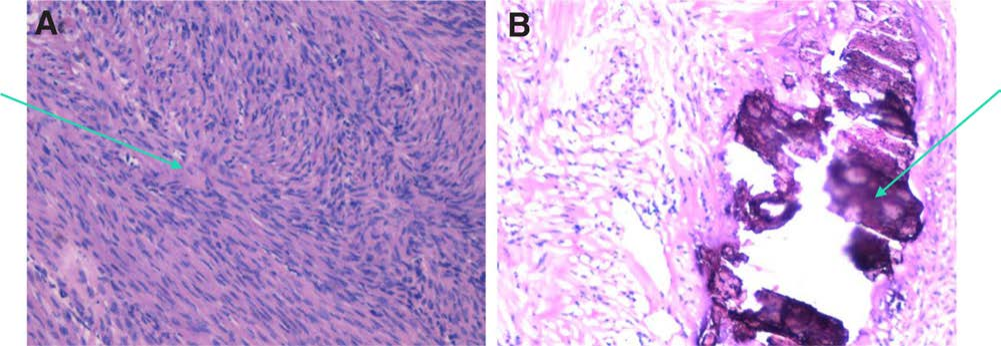
Intraoperative frozen section histopathology. (A) Pathological characteristics of ovarian leiomyoma (arrow) (1 × 100); (B) Calcification foci (arrow) (1 × 200).

**Figure 4.**
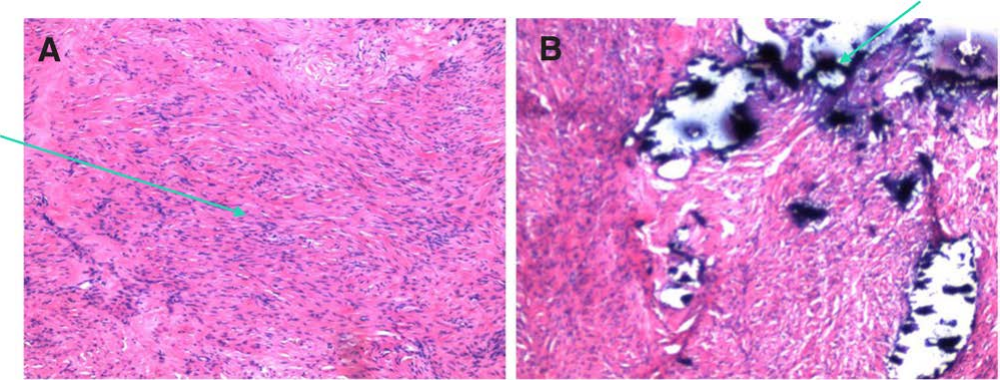
Postoperative histological results. (A) Positive staining for smooth muscle actin (1 × 100); (B) Calcification foci (arrow) (H&E, original magnification 1 × 200).

Intraoperative frozen section histopathology showed pathological characteristics of ovarian leiomyoma with calcification (Fig. 3). Postoperative pathological results showed ovarian smooth muscle cells with calcification (Fig. 4).

The patient was discharged home 3 days after surgery. At the most recent follow-up (5 months after operation) of the patients, ultrasound was performed and no abnormal echoes were suggested in the adnexal region.

## 3. Discussion

Most patients with ovarian leiomyoma are asymptomatic and may inadvertently present as a lower abdominal mass or adnexal masses when checked for other disorders. Some patients may have abdominal pain, heavy or low menstrual bleeding, frequent urination and other symptoms such as ascites, hydrothorax, and hydronephrosis.^[1,4,5]^ Gynecological examination can touch a round or oval smooth, mobile, non-tender medium-sized solid mass in the unilateral or bilateral adnexal area.^[5]^ Gynecological ultrasound cannot determine the nature of the tumor, but can only show low and moderate strong echo mixed masses.^[6]^ Sometimes it can be misdiagnosed as teratoma due to factors such as calcification, and CT is nonspecific.^[5,6]^

Ovarian leiomyomas share the same histological classification as uterine leiomyomas, and the current pathological diagnostic criteria for ovarian leiomyoma are based on those used for uterine leiomyomas.^[7]^ The diagnosis of primary ovarian leiomyoma adheres to the following principles. First of all, metastasis, dissemination, and involvement of extraovarian smooth muscle tumors must be excluded first. Second, microscopic examination confirms that it is consistent with the histological characteristics of leiomyoma.^[8]^ Immunohistochemistry confirms that smooth muscle differentiation of ovarian leiomyoma is obvious, with clear boundaries, homogeneous texture, and the tumors are round or oval in size, most of which are 4 to 5 cm in diameter. With the naked eye, ovarian leiomyoma has clear boundaries and homogeneous texture, and the tumor is round or oval, with different sizes, most of which are 4 to 5 cm in diameter.^[7,8]^ Under light microscopy, it is mostly circular or fusiform of uniform size, weave-like, sac-like, swirling arrangement, cytoplasmic eosinophilia, nucleus rod-like or round-like, located in the center, blunt at both ends, uniform chromatin, inconspicuous nucleoli, no rare nuclear fission (<1/10 HPF), none of these morphological features support malignancy. Certain cytotype, active nuclear divisions, and singular nuclei can also occur in the ovaries, such as ovarian atypical leiomyomas. Therefore, microscopic features can be used to distinguish it from leiomyosarcoma. In addition, primary ovarian leiomyoma is stained for combined SMA, Desmin, and α-inhibin, which helps distinguish it from sex cordstromal tumors of spindle cell components.

The treatment for patients with ovarian leiomyoma should be individualized based on the patients’ age. For patients with fertility requirements who can see fibroids and ovaries, ovarian tumor exfoliation should be considered, while in cases where fertility is not a concern and the patient is of advanced age, adnexectomy on the affected side may be required.

## 4. Conclusion

Our case highlights the significance of microscopic features in diagnosing primary ovarian leiomyoma, as it proves to be an effective approach for distinguishing primary ovarian leiomyoma from ovarian fibroma, leiomyosarcoma, and stromal tumors. Furthermore, individualized treatment should be considered based on the patient age and fertility needs.

## Author contributions

**Conceptualization:** Dajiang Lu.

**Formal analysis:** Dajiang Lu.

**Investigation:** Dajiang Lu, Hua Chen, Chengfang Yang.

**Project administration:** Yongguo Zhang.

**Validation:** Yanzhen Liu.

**Visualization:** Haishan Mo.

**Writing – original draft:** Dajiang Lu.

**Writing – review & editing:** Dajiang Lu.
